# Glucose level determines excitatory or inhibitory effects of adiponectin on arcuate POMC neuron activity and feeding

**DOI:** 10.1038/srep30796

**Published:** 2016-08-09

**Authors:** Shigetomo Suyama, Fumihiko Maekawa, Yuko Maejima, Naoto Kubota, Takashi Kadowaki, Toshihiko Yada

**Affiliations:** 1 Division of Integrative Physiology, Department of Physiology, Jichi Medical University School of Medicine, 3311-1 Yakushiji, Shimotsuke, Tochigi, 320-0498, Japan; 2 Molecular Toxicology Section, Center for Environmental Health Sciences, National Institute for Environmental Studies, Onogawa, Tsukuba, Ibaraki 305-8506, Japan; 3 Department of Electrophysiology and Oncology, Fukushima Medical University School of Medicine, 1 Hikarigaoka, Fukushima 960-1295, Japan; 4 Department of Diabetes and Metabolic Diseases, University of Tokyo Graduate School of Medicine, 7-3-1 Hongo, Bunkyo-ku, Tokyo 113-8655, Japan; 5 Division of Adaptation Development, Department of Developmental Physiology, National Institute for Physiological Sciences, Okazaki, Aichi 444-8585, Japan

## Abstract

Adiponectin regulates glucose and lipid metabolism, acting against metabolic syndrome and atherosclerosis. Accumulating evidence suggest that adiponectin acts on the brain including hypothalamic arcuate nucleus (ARC), where proopiomelanocortin (POMC) neurons play key roles in feeding regulation. Several studies have examined intracerebroventricular (ICV) injection of adiponectin and reported opposite effects, increase or decrease of food intake. These reports used different nutritional states. The present study aimed to clarify whether adiponectin exerts distinct effects on food intake and ARC POMC neurons depending on the glucose concentration. Adiponectin was ICV injected with or without glucose for feeding experiments and administered to ARC slices with high or low glucose for patch clamp experiments. We found that adiponectin at high glucose inhibited POMC neurons and increased food intake while at low glucose it exerted opposite effects. The results demonstrate that glucose level determines excitatory or inhibitory effects of adiponectin on arcuate POMC neuron activity and feeding.

Adipokines are the adipose tissue-derived hormones. Among them, leptin plays an important role in regulating energy metabolism and body weight. Adiponectin, another major adipokine, regulates glucose and lipid metabolism, and acts against metabolic syndrome and atherosclerosis^1^,^2^,^3^. Accumulating evidence also suggest that adiponectin acts on the central nervous system (CNS). Peripheral adiponectin could penetrate the cerebrospinal fluid (CSF) from circulation^4^. The adiponectin receptors, AdipoR1/2, are located in the brain including hypothalamus^4^,^5^. Adiponectin regulates neuronal activity in the paraventricular nucleus (PVN) of the hypothalamus and the nucleus of solitary tract of the brain stem^6^,^7^,^8^. Intracerebroventricular (ICV) injection of adiponectin has been shown to regulate food intake^4^,^9^, energy metabolism^4^,^10^, glucose metabolism^11^,^12^, bone metabolism^4^,^9^, and circulation systems^13^, and to induce phosphorylation of AMP-activated protein kinase (AMPK) pathway^4^, insulin receptor substrate (IRS)1/2–Akt–forkhead box protein O1 (FOXO1) pathway, and janus activating kinase 2 (JAK2)-signal transducer and activator of transcription 3 (STAT3) pathway^9^ via AdipoR1 in the mediobasal hypothalamus.

The proopiomelanocortin (POMC)-expressing neurons in the arcuate nucleus (ARC) are activated by hormonal and nutritional satiety signals such as leptin and glucose, which reflect systemic energy states^14^,^15^,^16^. POMC neurons, once activated, release α-melanocyte stimulating hormone, which interacts with melanocortin receptor 3/4, leading to suppression of food intake and promotion of energy expenditure^17^,^18^. AdipoR1/2, the adiponectin receptors, are located in the ARC POMC neurons^4^,^5^. These findings raise an important question whether adiponectin could regulate ARC POMC neurons.

The effect of ICV injection of adiponectin on food intake has been investigated. In one report, ICV injection of adiponectin increased food intake for 6 hr following 3 hr refeeding condition^4^. In another report, ICV adiponectin injection at late light phase decreased food intake during dark phase^9^. Thus, apparently opposite effects of ICV adiponectin on food intake have been observed. These reports used different timing of adiponectin injection, either before or during eating, which is associated with different metabolic states. Feeding-associated changes in nutritional and hormonal states are known to influence brain functions.

In the present study, we hypothesized that adiponectin could exert distinct effects on the central feeding center including ARC POMC neurons depending on nutritional and hormonal states. Among them, we focused on the glucose concentration, which regulates activities of both glucose-responsive and glucose-inhibited neurons in the brain including ARC^19^,^20^,^21^. Adiponectin was injected ICV with or without glucose to mimic fed or fasted states, respectively, and examined the effect on food intake and POMC neuron activity in mice. We found that ICV injection of adiponectin without or with glucose exerted reciprocal effects on the POMC neuron activity and feeding.

## Results

### ICV adiponectin injection glucose-dependently reciprocally regulates food intake

**Figure 1:**
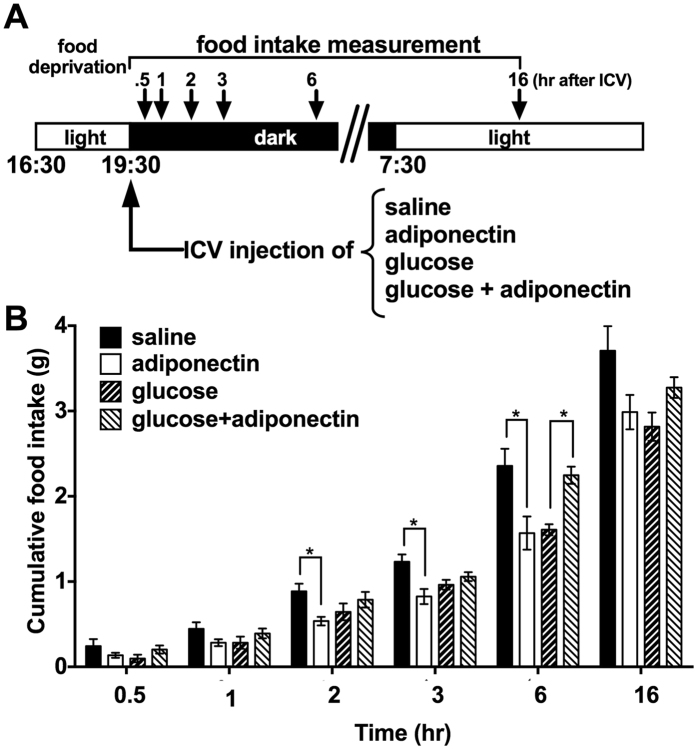
Central adiponectin suppressed or promoted food intake in glucose dependent manner. (**A**) Protocol for food intake measurement after ICV injection of adiponectin and/or glucose. (**B)** Cumulative food intake after ICV injection of saline or adiponectin with or without glucose at the end of light phase following 3 hr fasting (n = 6~12).*p < 0.05 (unpaired t-test).

Previous studies used different experimental conditions, including animal species and timing of adiponectin injection^4^,^9^. In the present study, mouse hexametric or middle molecular weight (MMW, 180 kDa) adiponectin (150 ng) or saline was ICV injected at the end of light phase (19:30) after fasting for 3 hr (Fig. 1A), following the procedure reported by Coope *et al*.^9^. This condition of 3 hr fasting at late light phase is considered to produce a moderate appetite, though it is milder than the intense starvation produced by overnight or 24 hr fasting. On the other hand, to produce a condition with considerable satiety, the same time course and food-deprived procedure were used but glucose (100 μg) was ICV injected together with adiponectin (Fig. 1A).

Glucose injection decreased food intake (Fig. 1B). ICV injection of adiponectin together with glucose significantly increased cumulative food intake at 6 hr after injection (glucose: 1.61 ± 0.06 g, glucose + adiponectin: 2.25 ± 0.10 g, p < 0.05, Fig. 1B). This result indicated that adiponectin increased food intake in the condition with elevated glucose in CNS.

After 3 hr fasting, a condition considered to lower the glucose concentration in the brain, single injection of adiponectin, compared to saline injection, significantly (p < 0.05) decreased food intake at 2, 3 and 6 hr after injection (Fig. 1B). This result demonstrated that adiponectin decreased food intake in the condition with low glucose in CNS, in consistent with previous study in rats^9^. This also indicated that species difference is not observed in the adiponectin effect on food intake.

### Adiponectin at high glucose hyperpolarizes hypothalamic ARC POMC neurons via AMPK

**Figure 2:**
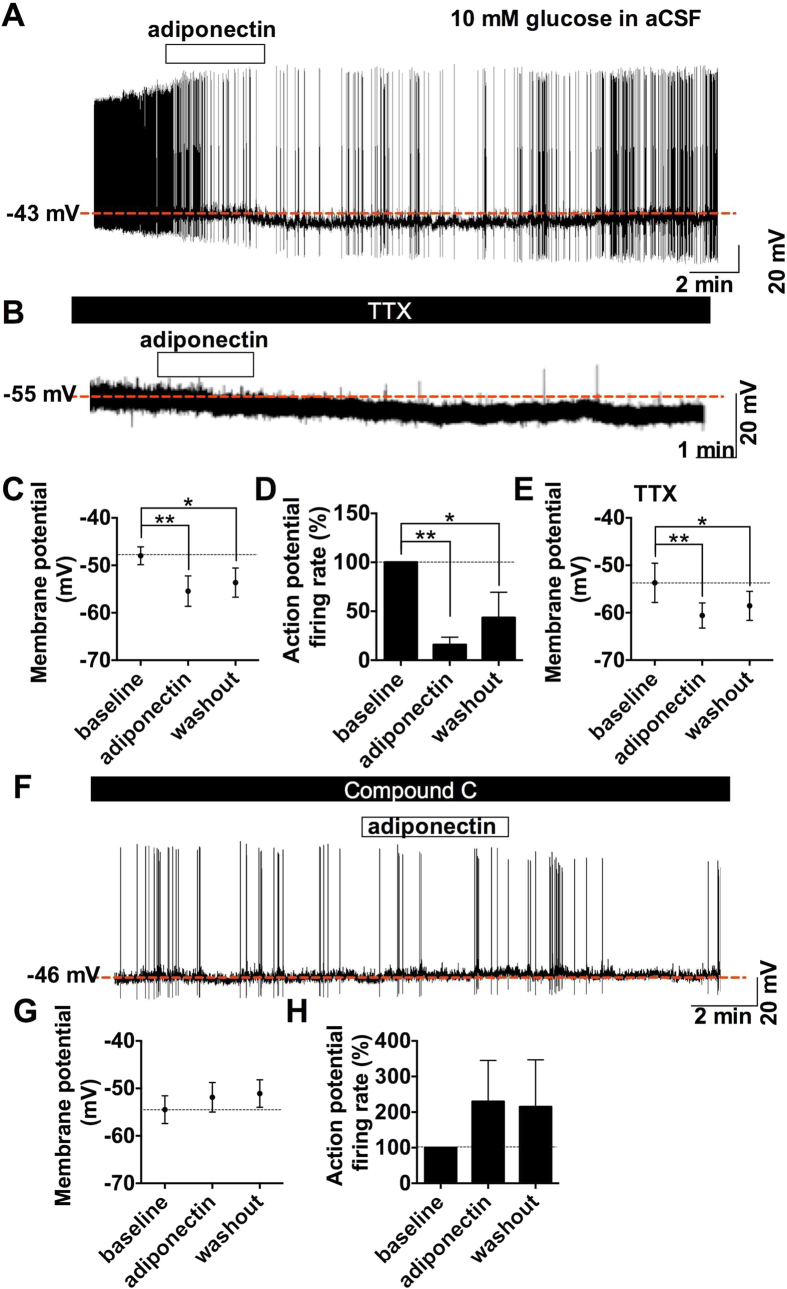
Adiponectin hyperpolarized POMC neurons via AMPK under high glucose condition. (**A,B)** Representative trace of current clamp recording with 10 mM glucose in aCSF in the absence (**A**) or presence (**B**) of TTX. **(C,D**) Membrane potential (**C**, n = 9) and action potential firing rate normalized by baseline (**D**, n = 9) before, during and after application of 0.56 nM adiponectin. (**E**) Membrane potential before, during and after application of adiponectin in the presence of 1 μM TTX. (**F**) Representative trace of current clamp recording with 10 mM glucose and 30 μM Compound C in aCSF. (**G,H**) Membrane potential (**G**, n = 6) and action potential firing rate normalized by baseline (**H**, n = 5) before, during and after administration of adiponectin in the presence of Compound C.

To investigate whether adiponectin regulates the activity of POMC neurons in ARC, membrane potentials and action potentials of ARC POMC neurons in the acute hypothalamic slice isolated from POMC-hrGFP transgenic mice were recorded by patch clamp experiments under whole-cell current clamp mode. In the high glucose condition, artificial CSF (aCSF) contained 10 mM glucose. Glucose at this concentration inhibits glucose-inhibited neurons implicated in stimulation of food intake^20^,^22^ and activates glucose-responsive neurons implicated in inhibition of food intake^23^. Bath application of hexametric form adiponectin (0.56 nM) for 4 min hyperpolarized the membrane potential from −48.0 ± 1.9 mV to −55.4 ± 3.2 mV (Fig. 2A,C) in nine of 12 POMC neurons (75%) and significantly decreased firing rate of action potential (Fig. 2A,D). The hyperpolarization continued to be observed at least for 20 min after washout of adiponectin. In the presence of 1 μM tetrodotoxin (TTX) that prevents presynaptic action potentials, five of seven POMC neurons (70%) were hyperpolarized from −53.7 ± 4.1 mV to −60.6 ± 2.7 mV (Fig. 2B,E) by adiponectin. Thus, TTX altered neither the incidence nor the amplitude of adiponectin-induced hyperpolarization in POMC neurons, indicating that the hyperpolarization was not mediated by altered neuronal transmission onto POMC neurons. These results indicate that, in the presence of high (10 mM) glucose, adiponectin directly interacts with POMC neurons to suppress their electrical activity.

We examined possible involvement of AMPK in the downstream signal pathway of adiponectin in POMC neurons. Since adiponectin reportedly regulates AMPK via AdipoR1 in peripheral organs^24^,^25^,^26^,^27^ and also in mediobasal hypothalamus including ARC^4^, whole-cell current clamp was performed with AMPK inhibitor, Compound C (30 μM). Adiponectin application induced hyperpolarization in none of eight POMC neurons (0%) (baseline: −54.5 ± 2.9 mV, adiponectin: −51.9 ± 3.1 mV, Fig. 2F,G). These results indicate that adiponectin under 10 mM glucose condition inhibits POMC neurons via a mechanism involving AMPK. These results suggest that adiponectin at high glucose may inactivate POMC neurons and increase food intake. These results are consistent with previous report that ICV adiponectin injected under conditions with mild satiety increases food intake.

### Adiponectin at low glucose depolarizes hypothalamic ARC POMC neurons via phosphoinositide 3-kinase (PI3K)

**Figure 3:**
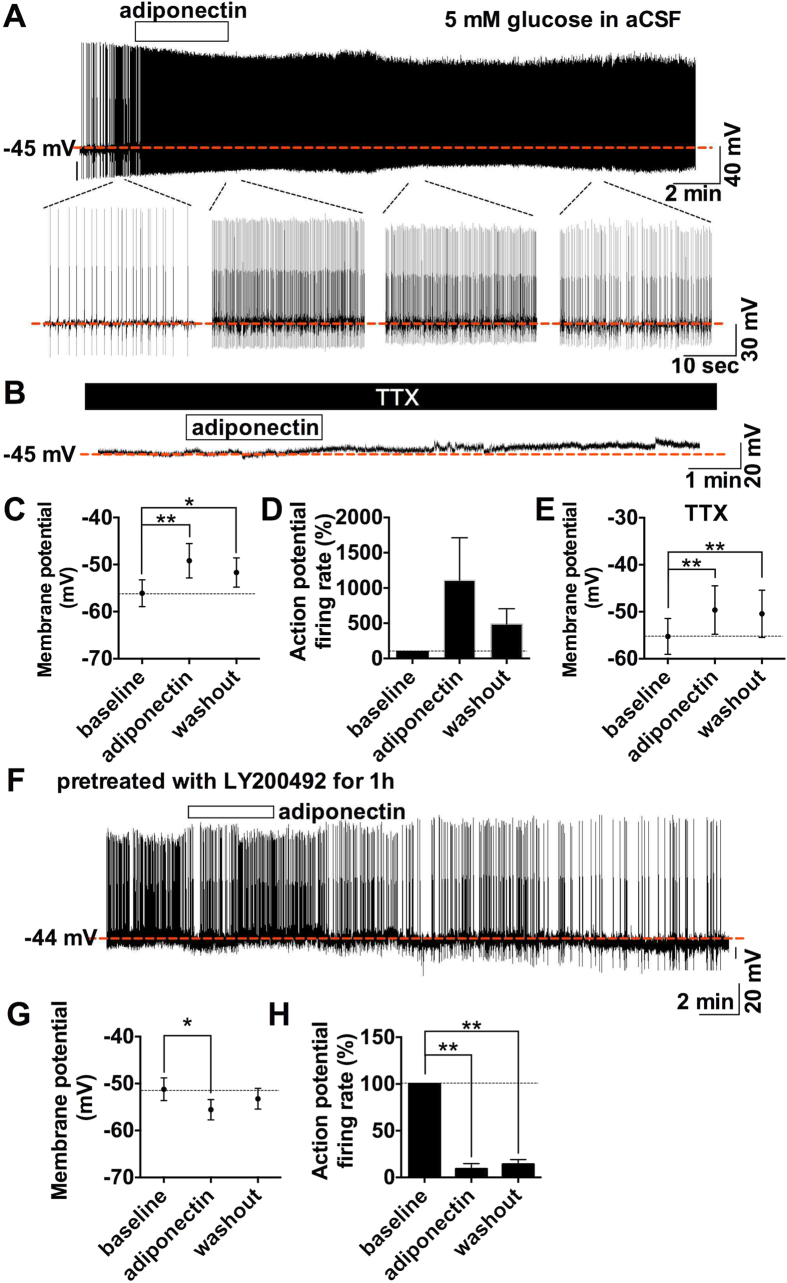
Adiponectin depolarized POMC neurons via PI3K pathway under lower glucose condition. (**A,B)**, Representative trace of current clamp recording with 5 mM glucose in aCSF in the absence (**A**) or presence (**B**) of 1 μM TTX. (**C,D**), Membrane potential (**C**, n = 8) and action potential firing rate normalized by baseline (**D**, n = 5) before, during and after administration of 0.56 nM adiponectin. (**E**) Membrane potential before, during and after administration of adiponectin in the presence of TTX (n = 5). (**F**) Representative trace of current clamp recording with 10 mM glucose in aCSF with the pretreatment of 50 μM LY200492, PI3K inhibitor. (**G,H**) Membrane potential (**G**, n = 8) and action potential firing rate normalized by baseline (**H**, n = 4)) before, during and after administration of adiponectin with the pretreatment of LY200492.

Current clamp recording was performed with lower glucose concentration of 5 mM in aCSF. Surprisingly, adiponectin application depolarized eight of 12 POMC neurons (67%) from −56.1 ± 2.9 mV to −49.2 ± 3.7 mV (Fig. 3A,C), while none of neurons were hyperpolarized. Action potential firing rate tended to increase in response to adiponectin (p = 0.08) (Fig. 3A **expanded scale and** 3D). In the presence of TTX, five of seven POMC neurons (71%) were depolarized from −55.3 ± 3.8 mV to −49.6 ± 5.1 mV (Fig. 3B,E). The incidence and amplitude of adiponectin-induced depolarization in POMC neurons were similar without and with TTX (Fig. 3C vs. 3E), indicating that the depolarization was not mediated by neuronal transmission onto POMC neurons. In the presence of 2.5 mM glucose in aCSF, adiponectin depolarized seven of 12 POMC neurons (58%) from −50.4 ± 2.5 mV to −40.9 ± 2.6 mV (Supplemental Fig. 1). Thus, similar results were obtained with 5 and 2.5 mM glucose. These data indicate that adiponectin directly interacts with POMC neurons to increase their electrical activity at low glucose, being consistent with the report by Coope *et al*.^9^ When POMC neurons were pretreated with PI3K inhibitor LY200492 (50 μM) for 1 hr, adiponectin failed to depolarize them and, instead, rather induced mild hyperpolarization (Fig. 3F) and reduced firing rates in some POMC neurons (Fig. 3F,H). This result is in consistent with previous report that adiponectin stimulates IRS1/2–Akt–FOXO1 pathway in mediobasal hypothalamus including ARC^9^. These results suggest that adiponectin depolarizes POMC neurons via a mechanism involving PI3K pathway.

## Discussion

We have demonstrated that ICV injection of adiponectin increases food intake when the glucose level in the brain is high, whereas it suppresses food intake when the glucose level in the brain is low. In parallel, adiponectin decreases the POMC neuron activity at high glucose (10 mM), whereas it increases the POMC neuron activity at low glucose (2.5~5 mM). Thus, adiponectin alters the ARC POMC neuron activity and food intake toward opposite directions depending on the energy state or glucose concentration in the brain. These results reveal level determines excitatory or inhibitory effects of adiponectin on the ARC POMC neuron activity and feeding. Furthermore, the current data suggest that adiponectin inhibits POMC neurons at high glucose via signaling involving AMPK (Fig. 2), while it activates POMC neurons at low glucose via signaling involving PI3K (Fig. 3). These signaling pathways may serve to couple adiponectin receptor to POMC neuron activity. The energy state- or glucose-dependent dual effects of adiponectin on POMC neurons with distinct signaling cascades may solve the apparent discrepancy in previous reports conducted under different nutritional states^4^,^9^.

Our data suggest a possible link of the adiponectin reception to AMPK and PI3K signaling in ARC neurons. This is in agreement with earlier studies. AdipoR1-derived signaling pathway includes AMPK in ARC neurons as well as in peripheral organs such as the liver and skeletal muscle^24^,^25^,^26^,^27^. Activation of AMPK hyperpolarizes POMC neurons^28^. Under conditions with low CSF glucose in the late light phase or after moderate fasting, ICV adiponectin injection phosphorylates IRS2-PI3K-Akt pathway in the mediobasal hypothalamus in rats^9^ and Lep^ob/ob^ mice^11^. Activation of PI3K-Akt pathway by leptin^29^ and insulin^30^,^31^ depolarizes POMC neurons via TRPC^32^,^33^. The leptin activation is blunted in POMC neurons deleted PDK1, a signaling molecule downstream of PI3K-Akt^34^,^35^.

**Figure 4:**
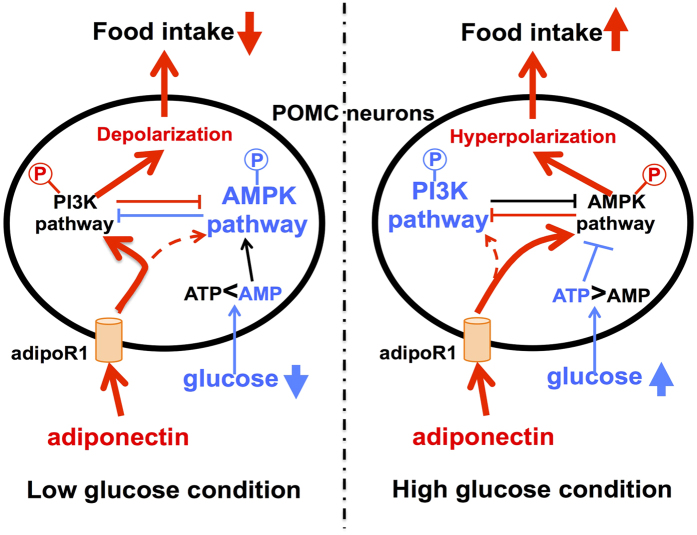
Hypothetical model of central adiponectin effect on POMC neurons and food intake. Under higher glucose condition, high level of ATP suppresses AMPK pathway, which leads to promotion of PI3K pathway. Adiponectin stimulate AMPK, but not PI3K pathway that is already activated. Activation of AMPK hyperpolarizes POMC neurons, and thereby promotes food intake. Under lower glucose condition, high level of AMP activate AMPK pathway and inactivate PI3K pathway. Adiponectin stimulate PI3K pathway, but not AMPK pathway which is already activated. Activation of PI3K pathway depolarizes POMC neurons, and thereby suppresses food intake. Red arrow/line indicates adiponectin-stimulated pathway.

In the presence of AMPK inhibitor, adiponectin at high glucose failed to hyperpolarize but tended to depolarize POMC neurons, showing the reversal of the adiponectin effect (Fig. 2G,H). Conversely, after pretreatment with PI3K inhibitor, adiponectin at low glucose failed to depolarize but moderately hyperpolarized POMC neurons, showing a reversed effect (Fig. 3G,H). These results suggest that adiponectin simultaneously induces two signaling pathways that function toward opposite directions in POMC neurons. It is suggested that at high glucose, adiponectin activation of PI3K pathway is less influential than that of AMPK pathway but becomes obvious when AMPK pathway is inhibited. Likewise, at low glucose, adiponectin activation of AMPK pathway is less influential than that of PI3K pathway but becomes obvious when AMPK pathway is inhibited. Additionally, previous studies suggest that AMPK and PI3K pathways may act competitively or reciprocally. Constitutive active form of AMPK suppressed the leptin pathway involving PI3K^36^. AMPK binds and phosphorylates ^794^Ser of IRS1, which may inhibit PI3K pathway^37^. Furthermore, AMPK phosphorylate TSC2 and Raptor which are suppressor of mTORC1^38^,^39^. On the other hand, Akt or mTOR-S6 kinase, downstream target of PI3K, phosphorylate ^485^Ser and ^491^Ser of α1AMPK, possibly suppressing AMPK activity^40^,^41^,^42^. These reports support that glucose-induced suppression of AMPK could activate PI3K pathway. Based on our present finding together with these previous reports, we propose that high vs. low glucose shifts the dominant signaling pathway between AMPK and PI3K, and that the yet poorly stimulated signaling pathway under each glucose concentration could be preferentially and fully activated by adiponectin, which results in glucose-dependent reciprocal effects of adiponectin on POMC neurons and food intake (Fig. 4). Under high glucose condition, PI3K in POMC neuron is already activated by high glucose, insulin and/or leptin, and adiponectin cannot influence it further but mainly activates AMPK pathway to hyperpolarize POMC neurons. Under low glucose condition, AMPK in POMC neuron is already phosphorylated by low glucose and associated increase of AMP/ATP ratio, and adiponectin cannot stimulate it further but chiefly activates PI3K pathway to depolarize POMC neurons. Based on these findings, we propose that adiponectin moderately counteracts with both high glucose and low glucose, serving as an attenuator of profound effect of glucose change on POMC neuron activity and feeding.

In current clamp recordings, recovery from adiponectin effect was not obtained till 15~20 min after its washout. The reason may be the sticky nature of adiponectin due to its collagen domain^43^. Therefore, we could not test whether adiponectin glucose-dependently depolarize and/or hyperpolarize the same or distinct subpopulations of POMC neurons. However, the experiments with AMPK inhibitor or PI3K inhibitor showed that adiponectin can simultaneously induce dual effects on POMC neurons toward opposite directions (Figs 2G,H and 3G,H). This result suggest that adiponectin can stimulate both AMPK pathway and PI3K pathway on the same POMC neuron subpopulation and that the final effect is determined by the glucose-dependent balance between hyperpolarization partly medicated by AMPK and depolarization partly medicated by PI3K.

In the present study, ICV adiponectin co-injected with glucose did not significantly increase food intake in early phase (0~3 hr), while adiponectin rapidly inhibited the electrical activity of POMC neurons by current clamp recording. The mechanism underlying this apparent dissociation remains to be elucidated. However, we speculate that in *in vivo* experiment, since adiponectin and glucose were injected simultaneously, it may require time for adiponectin’s orexigenic effect to overcome the glucose-induced anorexigenic effect. On the other hand, *in vitro* electrical recording was performed in POMC neurons that had been exposed to 10 mM glucose for more than 1.5 hr, in which no acute effect of glucose was present, and hence the adiponectin administration could rapidly activate AMPK and hyperpolarize POMC neurons.

In the present study, we found glucose- or energy state-dependent opposing effects of adiponectin on POMC neuron activity and feeding. The physiological significance of this reciprocal action of adiponectin remains to be clarified. However, we can speculate its possible role from the point of adaptation and evolution. In the basal energy state, adiponectin suppresses food intake. However, once food is available, adiponectin stimulates food intake. Under wild circumstance with restricted food, it has been essential for living species to store more energy. This may be the reason why adiponectin stimulates food intake in positive energy states. Thus, adiponectin may have contributed to the adaptation and survival of living species, as well as regulation of metabolism. In fact, adiponectin and their receptor homolog have been highly conserved from yeast to human^44^,^45^.

## Methods

### Food intake measurement

Male C57black6/J mice aged 8–10 weeks were maintained in a 12/12 hrs light/dark cycle. For ICV injection in mice, a gauge guide cannula (type; OM205-113, diameter; 0.2 mm; Unique medical, Tokyo, Japan) was placed stereotaxically into 3V, at 1.5 mm caudal to the bregma in the midline and 5 mm below the surface of the skull, under anesthesia with tribromoethanol (200 mg/kg). Mouse was allowed to recover from the operation for 1 week while they were habituated to handling. On the day of experiments, food was removed form cages at 16:00. At 19:30, mouse received an injection of 150 μg mouse purified MMW-adiponectin^46^ without or with 100 μg glucose dissolved in vehicle (sterile saline; 0.9% NaCl). The food was returned to cages, and food intake at 0.5, 1, 2, 3, 6, 16 hr were measured. At the end of the experiments, sections of the hypothalamus were histologically examined to verify the position of the cannulas. The animal protocols for this study were approved by Jichi Medical University Institute of Animal Care and Use Committee., and all experiments were carried out in accordance with the approved protocols.

### Acute slice preparation

POMC-hrGFP transgenic mice aged 7–10 weeks were maintained in a 12/12 h light/dark cycle. Their brains were then removed rapidly from mouse anesthetized with tribromoethanol (200 mg/kg), and placed in an ice-cold, carboxygenated (95% O_2_ and 5% CO_2_) high mannitol solution that contained (in mM) 229 mannitol, 3 KCl, 6 MgCl_2_, 0.5 CaCl_2_, 1 NaH_2_PO_4_, 26 NaHCO_3_, and 10 glucose, pH7.4 (with an osmolarity of 300–305 mOsm) with 0.5 μM tetrodotoxin. A block of tissue containing the hypothalamus was isolated and coronal slices (300 μm) were cut on a Vibratome. After a 1–2 hrs recovery period, slices were moved to a recording chamber mounted on a BX51WI upright microscope (Olympus) equipped with video-enhanced infrared-differential interference contrast (DIC) and fluorescence. Slices were perfused with a continuous flow of carboxygenated aCSF that contained (in mM) 127 NaCl, 2.5 KCl, 2 MgCl_2_, 2 CaCl_2_, 1.23 NaH_2_PO_4_, 26 NaHCO_3_, and 2.5–10 glucose, pH 7.4 Neurons were visualized with an Olympus Optical 40x water-immersion lens.

### Patch-clamp recording

Whole-cell current-clamp recordings were performed as previously reported^47^ Briefly, pipettes were used with 3–9 MΩ resistance after being filled with pipette solution. Pipettes were made of borosilicate glass (Narishige) using a PP-83 vertical puller (Narishige) or a Sutter micropipette puller (P-1000). The pipettes were used with 3–9 MΩ resistance after being filled with pipette solution. The composition of the pipette solution was as follows (in mM): 135 K-gluconate, MgCl_2_ 2, HEPES 10, EGTA 1.1, Mg-ATP 2.5, Na_2_-GTP 0.3, and Na_2_-phosphocreatine 10, pH 7.3 with KOH (with an osmolarity of 290–295 mOsm). An Axopatch 200B amplifier and Clampex 9.2 or 10 software (Axon Instruments) were used for data acquisition. Pclamp 9.2 or 10 (Axon Instruments) software was used for analysis. Liquid junction potential correction was performed off-line. Access resistance was continuously monitored during the experiments. Only those cells in which access resistance was stable (changes ~30%) were included in the analysis. The data was analyzed by Clamp fit 9.2 or 10 (Axon instruments) software and GraphPad Prism6 software. The response by adiponectin was defined as a change over 2 times standard deviation of membrane potential for 2 min before applying adiponectin.

### Statistical Analysis

One-way ANOVA followed by Dunnet multiple range tests were used to compare multiple test groups and unpaired Student’s t tests were used for two groups.

## Additional Information

**How to cite this article**: Suyama, S. *et al*. Glucose level determines excitatory or inhibitory effects of adiponectin on arcuate POMC neuron activity and feeding. *Sci. Rep.* **6**, 30796; doi: 10.1038/srep30796 (2016).

## Supplementary Material

Supplementary Information
