# Supplementary material for: Glucose level determines excitatory or inhibitory effects of adiponectin on arcuate POMC neuron activity and feeding
Source: Sci Rep. 2016 Aug 9;6:30796. doi: 10.1038/srep30796 (PMC4977585; doi:10.1038/srep30796)
Supplement: Supplementary Information [file srep30796-s1.pdf]

# Glucose level determines excitatory or inhibitory effects of adiponectin on arcuate POMC neuron activity and feeding

Shigetomo Suyama[1], Fumihiko Maekawa[2], Yuko Maejima[3], Naoto Kubota[4], Takashi Kadowaki[4] and Toshihiko Yada [1, 5,†]

[1] Division of Integrative Physiology, Department of Physiology, Jichi Medical University School of Medicine, 3311-1 Yakushiji, Shimotsuke, Tochigi, 320-0498, Japan

[2] Molecular Toxicology Section, Center for Environmental Health Sciences, National Institute for Environmental Studies, Onogawa, Tsukuba, Ibaraki 305-8506, Japan.

[3] Department of Electrophysiology and Oncology, Fukushima Medical University School of Medicine, 1 Hikarigaoka, Fukushima 960-1295, Japan.

[4] Department of Diabetes and Metabolic Diseases, University of Tokyo Graduate School of Medicine, 7-3-1 Hongo, Bunkyo-ku, Tokyo 113-8655, Japan.

[5] Division of Adaptation Development, Department of Developmental Physiology, National Institute for Physiological Sciences, Okazaki, Aichi 444-8585, Japan

[†] Corresponding author

Correspondence to:
Prof. Toshihiko Yada
Division of Integrative Physiology, Department of Physiology, Jichi Medical University School of Medicine, 3311-1, Yakushiji, Shimotsuke, Tochigi, 329-0498, Japan.
Tel: +81-285-58-7319
Fax: +81-285-44-9962
E-mail: tyada@jichi.ac.jp

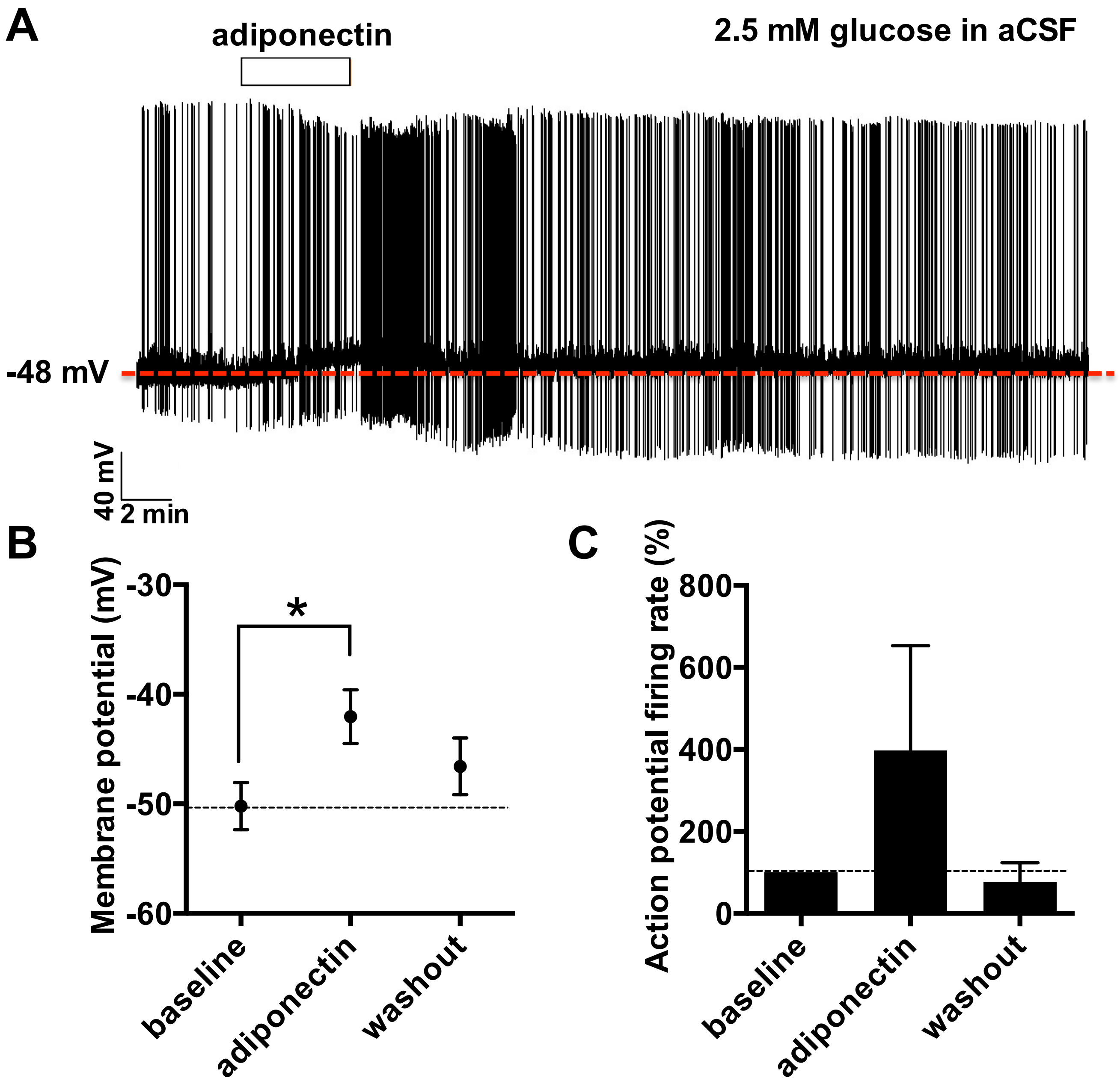

A
adiponectin
2.5 mM glucose in aCSF
-48 mV
40 mV
2 min
B
Membrane potential (mV)
-30
-40
-50
-60
*
baseline
adiponectin
washout
C
Action potential firing rate (%)
800
600
400
200
0
baseline
adiponectin
washout

**Supplemental figure 1.** Adiponectin depolarized POMC neurons under 2.5 mM glucose condition.

**A,** Representative trace of current clamp recording with 2.5 mM glucose in aCSF. **B and C,** Membrane potential (B, n=7) and action potential firing rate normalized by baseline (C, n=3) before, during and after administration of adiponectin.
